# Outcome of patients with diffuse large B-cell lymphoma and testicular involvement – real world data

**DOI:** 10.1007/s00277-024-06025-y

**Published:** 2024-10-01

**Authors:** Heidi Mocikova, Andrea Janikova, Alice Sykorova, Vit Prochazka, Jan Pirnos, Juraj Duras, Katerina Kopeckova, Katerina Steinerova, Robert Pytlik, Petra Blahovcova, David Salek, Tomas Kozak, Veronika Bachanova, David Belada

**Affiliations:** 1 Fakultni nemocnice Kralovske Vinohrady, Department of Haematology and Third Faculty of Medicine, Charles University, Prague, Czech Republic; 2 Department of Internal Medicine, Hematology and Oncology, Faculty of Medicine, University Hospital Brno, Masaryk University Brno, Brno, Czech Republic; 3 University Hospital and Faculty of Medicine, 4th Department of Internal Medicine– Hematology, Hradec Kralove, Czech Republic; 4 Faculty of Medicine and Dentistry, Department of Haemato-Oncology, Palacky University, Olomouc, Czech Republic; 5 Ceske Budejovice Hospital, Ceske Budejovice, Czech Republic; 6 University Hospital and Faculty of Medicine, Department of Hemato-Oncology, Ostrava, Czech Republic; 7 Department of Oncology of the 2nd Faculty of Medicine of Charles University, Motol University Hospital, Prague, Czech Republic; 8 Department of Clinical Hematology, University Hospital, Pilsen, Czech Republic; 9 Cell Therapy Department, Institute of Haematology and Blood Transfusion, Prague, Czech Republic; 10 Datacenter of the Czech Lymphoma Study Group, Prague, Czech Republic; 11 Division of Hematology, Oncology and Transplantation, University of Minnesota, Minneapolis, MN USA

**Keywords:** Diffuse large B-cell lymphoma, Testicular involvement, Orchiectomy, Rituximab, CNS relapse

## Abstract

Patients with testicular lymphoma are at an increased risk of central nervous system (CNS) disease. Optimal strategy for CNS relapse prevention is unknown. We analyzed treatment strategies, cumulative incidence of CNS relapse and prognosis in 229 patients with diffuse large B-cell lymphoma (DLBCL) and testicular involvement: 157 primary testicular lymphomas (PTL) in clinical stages IE/IIE and 72 patients in advanced stages (T-DLBCL) IIIE/IV. Treatments for PTL vs. T-DLBCL included: rituximab-based chemotherapy (80.9% vs. 90.3%), orchiectomy (94.3% vs. 65.3%) and contralateral testicular irradiation (59.8% vs. 44.4%). Majority (84.3%) received CNS prophylaxis with similar rates of prophylactic methotrexate (intravenous 19.1% vs. 16.6%, intrathecal 40.8% vs. 40.4%, or both 24.2% vs. 27.8%) between PTL and T-DLBCL (*p* = 0.89). Median follow-up was 51.8 months. CNS relapses occurred in 14 (6.1%) of 63 relapsing patients. The 5-year cumulative incidence of CNS relapse in PTL was 4.5% and in T-DLBCL 12.1%. Median time to CNS relapse was 21.9 months. In univariate analyses, orchiectomy was the single significant factor associated with lower risk of CNS relapse in PTL (HR = 0.11 [95% CI, 0-0.124], *p* = 0.001). Rituximab significantly reduced CNS relapse risk in T-DLBCL (HR = 0.1002, *p* = 0.0005). Median progression-free survival (PFS) and overall survival (OS) following CNS relapse was dismal in T-DLBCL compared to PTL (PFS 1.6 vs. 37.8 months, *p* = 0.04 and OS 2.3 vs. 37.8 months, *p* = 0.05). This study confirmed a favorable impact of rituximab in prevention of CNS relapse in T-DLBCL. Methotrexate prophylaxis did not alter CNS relapse risk. Prognosis of CNS relapse is particularly poor in T-DLBCL.

**Supplementary Information:**

The online version contains supplementary material available at 10.1007/s00277-024-06025-y.

## Introduction

PTL represents 1–2% of all non-Hodgkin lymphomas. Median age at diagnosis is 67 years, although more than a quarter of the patients are younger than 60 years and more than 13% are younger than 50 years [1]. PTL is considered as an aggressive B-cell lymphoma of immune-privileged sites concurrently with the primary CNS lymphoma and the vitreoretinal lymphoma according to WHO-HAEM5 classification [2]. The most common histology in PTL is DLBCL with activated B-cell-like (ABC) or non-germinal center B-cell-like (non-GCB) phenotype comprising up to 90% of these cases [3, 4]. Mutational profile in lymphomas of immune-privileged sites includes concomitant MYD88 and CD79B mutations with genomic signature C5/MCD/MYD88 and genetic inactivation of MHC class I and II and β2-microglobulin with subsequent loss of protein expression [2]. NF-κB pathway signaling, 9p24.1 aberrations, and tumor-infiltrating immune cells, especially immune checkpoint expressing lymphocytes and macrophages are involved in pathogenesis of PTL [5]. Rearrangements of CD274 and PDCD1LG2 (coding programmed cell death ligands 1 and 2, PD-L1 and PD-L2) and BCL6 have been associated with increased risk of CNS relapse, and mutations of TBL1XR1 and overexpression of p53 with inferior outcome [6–8]. Retrospective analyses from the SEER database identified five risk factors affecting the prognosis of PTL in immunocompetent patients (age, laterality, Ann Arbor stage, chemotherapy, and radiotherapy) [9, 10]. Yang et al. constructed a prognostic nomogram model for the assessment of their survival [10]. Leivonen et al. demonstrated, that poor survival of PTL correlated with low percentage of CD3 + CD4 + and CD3 + CD8 + tumor-infiltrating lymphocytes (*P* < 0.001) [11]. Testicular involvement occurs rarely as an extranodal site in advanced T-DLBCL and it was analyzed together with PTL in several retrospective studies [12–14]. The reported cumulative incidence of CNS relapse after R-CHOP (rituximab, cyclophosphamide, doxorubicin, vincristine, prednisone) treatment in PTL and advanced T-DLBCL is high (5-year 10% vs. 24% and 10-year 27% vs. 24%, respectively) [12]. The median time to CNS recurrence is longer in PTL compared to T-DLBCL (5.4 years vs. 0.5 years) and prognosis of PTL and T-DLBCL in CNS relapse remains poor. Irradiation (RT) of the contralateral testis in combination with anthracycline based chemotherapy [15] or with rituximab based chemotherapy improved survival in patients with testicular involvement [4, 16–18]. The addition of rituximab has reduced the risk of lymphoma recurrence, however, its impact on the risk of CNS relapse remains controversial [12, 13, 19]. Tumor-infiltrating lymphocytes and tumor-associated macrophages have a crucial role in inducing response to both chemotherapeutic agents and rituximab [20]. Patients with a high T-cell inflamed tumor microenvironment had a better response to rituximab-based immunochemotherapy, as compared to other patients and T-cell inflamed tumor microenvironment is associated with favorable survival in PTL [11]. The International Extranodal Lymphoma Study Group (IELSG)10 prospective phase 2 trial addressed feasibility and activity of 6–8 cycles of R-CHOP combined with 4 doses of intrathecal (i.t.) MTX 12 mg and prophylactic RT to the contralateral testis at 25 to 30 Gy in 53 pts with PTL [16]. The 5-year cumulative incidence of CNS relapse was 6% (95% CI, 0 − 12%). The subsequent multicenter phase 2 prospective trial IELSG30 trial evaluated PFS in 54 PTL treated with 6 cycles of R CHOP 21 combined with 4 doses of i.t. liposomal cytarabine of 50 mg; 2 cycles of MTX 1.5 g/m^2^ i.v.; and prophylactic RT to the contralateral testis of 25–30 Gy [21]. IELSG30 trial demonstrated the 5-year PFS and OS rates 91% and 92%, and no CNS relapses at a median follow-up of 6 years [21]. The optimal CNS prophylaxis especially in advanced DLBCL with testicular involvement is not yet defined and internationally accepted. We analyzed the cumulative incidence of CNS relapse, time to CNS relapse, PFS, OS and the impact of various treatment strategies on CNS relapse in pts with DLBCL and testicular involvement prospectively observed in the Czech Lymphoma Study Group Registry.

## Materials and methods

### Patients

All patients signed the written informed consent to enroll to the Czech Lymphoma Study Group Registry for data collection and analyses that were approved by the ethics committees in participating centers. This analysis was done according to the Declaration of Helsinki and guidelines of Good Clinical Practice. Patients with DLBCL at the age ≥ 18 years at diagnosis were included; their pathology reports were centrally reviewed by reference hematopathologists. Standard workup at diagnosis and at relapse included assessment of ECOG performance status, physical examination, laboratory studies, bone marrow biopsy, CT of neck, chest, abdomen and pelvis or PET/CT imaging. Testicular involvement was determined by diagnostic orchiectomy or by imaging methods (scrotal ultrasound, CT or PET/CT). Unilateral or bilateral testicular involvement in PTL patients was staged as a single extranodal site IE and additional regional nodal involvement was classified as IIE. Patients in clinical stages IIIE/IV with testicular involvement were considered as advanced T-DLBCL. Patients with suspected CNS involvement at diagnosis or at relapse underwent neurological and ophthalmologic examinations, magnetic resonance imaging (MRI) of the brain and/or the spine, analysis of cerebrospinal fluid (CSF) including cytology and flow cytometry, and brain biopsy if it was infiltrated. Cases with initial CNS involvement and/or HIV seropositivity were excluded from this analysis. Treatment response was evaluated by CT or PET/CT and in CNS relapse by brain MRI and/or CSF examination.

### Statistical analysis

Characteristics of patients were compared using the Fisher exact test or Chi-square test. Progression-free survival (PFS) was defined as the time interval from the day of diagnosis until the first objective evidence of relapse/progression or death from any cause. PFS2 was measured from the date of diagnosis of a relapse until the next relapse/progression or death from any cause. Cumulative incidence of CNS lymphoma relapse was measured from the date of diagnosis to the date of CNS lymphoma relapse. Overall survival (OS) was calculated from the date of diagnosis to the date of the last follow-up or the date of death from any cause. OS2 was defined as the time interval from the date of a relapse until the date of the last follow-up or the date of death from any cause. Survival curves were calculated by Kaplan–Meier survival analysis and a comparison between subgroups was performed by the log-rank test. Risk factor analysis (univariate and multivariate) was assessed by Cox proportional hazards regression. P values of less than 0.05 were considered statistically significant.

## Results

### Patients

We analyzed 229 patients with DLBCL and testicular involvement treated between 2000 and 2022: 157 PTL (clinical stages IE 111 and IIE 46) and 72 patients had T-DLBCL (clinical stages IIIE 17 and IV 55). Kidney involvement was confirmed in 17 patients and 43 cases had other extranodal involvement (excluding testis). T-DLBCL represents 3% of all DLBCL in the Czech Lymphoma Study Group Project NiHiL: PTL (2%) and T-DLBCL (1%) and these data correspond well with previous reports [1, 12, 13]. Median age was 70 years (range 33–87). The non-GCB subtype was the most common 78/229 patients (34.1%) among identified subtypes of DLBCL, Table 1. Majority of patients had a good performance status at diagnosis ECOG 0–1(83%). Low IPI score (0–1 risk factor) was present in 111 (48%) patients, 86 (38%) patients had intermediate score (2–3 risk factors) and 32 (14%) patients had high score (4–5 risk factors), Table 1.

**Table 1:** Characteristics of patients

Characteristics	All patients	Primary testicular lymphoma	Advanced disease with testicular involvement	P
Number of patients	229 (100%)	157 (68.6%)	72 (31,4%)	-
Clinical stage	IE-IV (229)	IE/IIE (111/46)	IIIE/IV (17/55)	-
Age (median, range), years	70 (33–87)	70 (33–87)	70 (40–85)	0.77
DLBCL subtype				0.58
NOS	17 (7.4%)	13 (8.3%)	4 (5,6%)	
GC	33 (14.4%)	20 (12.7%)	13 (18.1%)	
Non GC	78 (34.1%)	52 (33.2%)	26 (36.1%)	
Unknown	101 (44.1%)	72 (45.8%)	29 (40.2%)	
B symptoms				< 0.0001
Yes	41 (17.9%)	13 (8.3%)	28 (38.9%)	
No	188 (82.1%)	144 (91.7%)	44 (61.1%)	
ECOG				0.0007
0–1	191 (83.4%)	140 (89.2%)	51 (70.8%)	
2–4	38 (16.6%)	17 (10.8%)	21 (29.2%)	
Elevated LDH				0.00003
Yes	88 (38.4%)	46 (29.3%)	42 (58.3%)	
No	141 (61.6%)	111 (70.7%)	30 (41.7%)	
IPI				< 0.0001
0–1	111 (48.5%)	107 (68.2%)	4 (5.6%)	
2	52 (22.7%)	44 (28.0%)	8 (11.1%)	
3	34 (14.8%)	6 (3.8%)	28 (38.9%)	
4–5	32 (14.0%)	0 (0.0%)	32 (44.4%)	
Kidney/adrenal involvement				< 0.0001
Yes	17 (7.4%)	0 (0.0%)	17 (23.6%)	
No	212 (92.6%)	157 (100%)	55 (76.4%)	
Other EN involvement (testicular excluded)				< 0.0001
Yes	43 (18.8%)	0 (0.0%)	43 (59.7%)	
No	186 (81.2%)	157 (100%)	29 (40.3%)	
Orchiectomy	195 (85.2)	148 (94.3%)	47 (65.3%)	< 0.0001
Prophylaxis	193 (84.3)	132 (84.1)	61 (84.7)	0.89
MTX i.t.	93 (40.6)	64 (40.8)	29 (40.4)	
MTX i.v.+/-ARA C	42 (18.3)	30 (19.1)	12 (16.6)	
MTX i.t.+i.v.	58 (25.3)	38 (24.2)	20 (27.8)	
Radiotherapy (contralateral testis)	126 (55.0)	94 (59.8)	32 (44.4)	0.07
No prophylaxis (i.v./i.t./without RT)	36 (15.7)	25 (15.9)	11 (15.3)	1.0
Rituximab + chemotherapy	192 (83.8)	127 (80.9)	65 (90.3)	0.0514
Chemotherapy	224 (97.8)	152 (96.8)	72 (100)	0.36
CHOP	217 (94.8)	147 (93.6)	70 (97.2)	
PACEBO	4 (1.8)	2 (1.3)	2 (1.8)	
HyperCVAD/MTX-ARA C	1 (0.4)	1 (0.6)	0	
DHAP/ESHAP	2 (0.9)	2 (1.3)	0	
No chemotherapy	5 (2.2)	5 (3.2)	0	

DLBCL, diffuse large B-cell lymphoma; NOS, not otherwise specified; GC, germinal center; LDH, lactate dehydrogenase; IPI, International Prognostic Index; EN, extranodal; MTX, methotrexate; i.t., intrathecal; i.v., intravenous; ns, not significant; RT, radiotherapy; CHOP, cyclophosphamide, doxorubicin, vincristine, prednisone; PACEBO, prednisone, doxorubicin, cyclophosphamide, etoposide, bleomycin, vincristine; hyper-CVAD/MTX-AraC, fractionated cyclophosphamide, vincristine, doxorubicin, dexamethasone, methotrexate, cytarabine; DHAP, cytarabine, cisplatin, dexamethasone; ESHAP, etoposide, cytarabine, cisplatin, methylprednisolone

### Treatment and CNS prophylaxis

Almost all patients (94.3%) underwent diagnostic orchiectomy: 148 (94.3%) patients with PTL and 47 (65.3%) with T-DLBCL (*p* < 0.0001). Bilateral orchiectomy was performed in only six patients. Rituximab in combination with chemotherapy was administered to 192 (83.8%) patients: 127 (80.9% of PTL) and 65 (90.3% of T-DLBCL). The regimen used included predominantly CHOP or CHOP-like regimens (PTL *n* = 147; 93.6% and T-DLBCL *n* = 70; 97.2%) followed by other less frequent regimens: PACEBO (prednisone, doxorubicin, cyclophosphamide, etoposide, bleomycin, vincristine; *n* = 4), hyper-CVAD/MTX-AraC (fractionated cyclophosphamide, vincristine, doxorubicin and dexamethasone alternating with high-dose methotrexate-cytarabine; *n* = 1), DHAP (high dose cytarabine, cisplatin, dexamethasone; *n* = 1) and ESHAP (etoposide, cytarabine, cisplatin, methylprednisolone, *n* = 1), Table 1. The median number of CHOP cycles or alternative chemotherapy regimens was 6 (range 1–8). Contralateral testis was irradiated in 94 patients with PTL (59.8%) and 32 patients with T-DLBCL (44.4%), *p* = 0.07, median dose was 30 Gy (range 15–54). RT was not used after bilateral orchiectomy. Overall 193 patients (84.3%) received CNS prophylaxis with MTX. There were similar rates in the use of MTX between PTL and T-DLBCL (*p* = 00.89): MTX i.t. 64 (40.8%) vs. 29 (40.3%), MTX i.v. with or without cytarabine 30 (19.1%) vs. 12 (16.6%); MTX combined i.t. and i.v. 38 (24.2%) vs. 20 (27.8%), respectively (Table 1). Treatment included intrathecal MTX (12 mg per dose) via lumbar puncture administered at median 4 doses (range 1–8) and intravenous MTX (median dose 3 g/m^2^ (range 1–3 g/m^2^) at median 2 doses (range 1–4). Out of 229 cases, about 15% did not recieve any CNS prophylaxis with no difference between PTL and T-DLBCL, *p* = 1.0).

### Relapses and survival

**Fig. 1:**
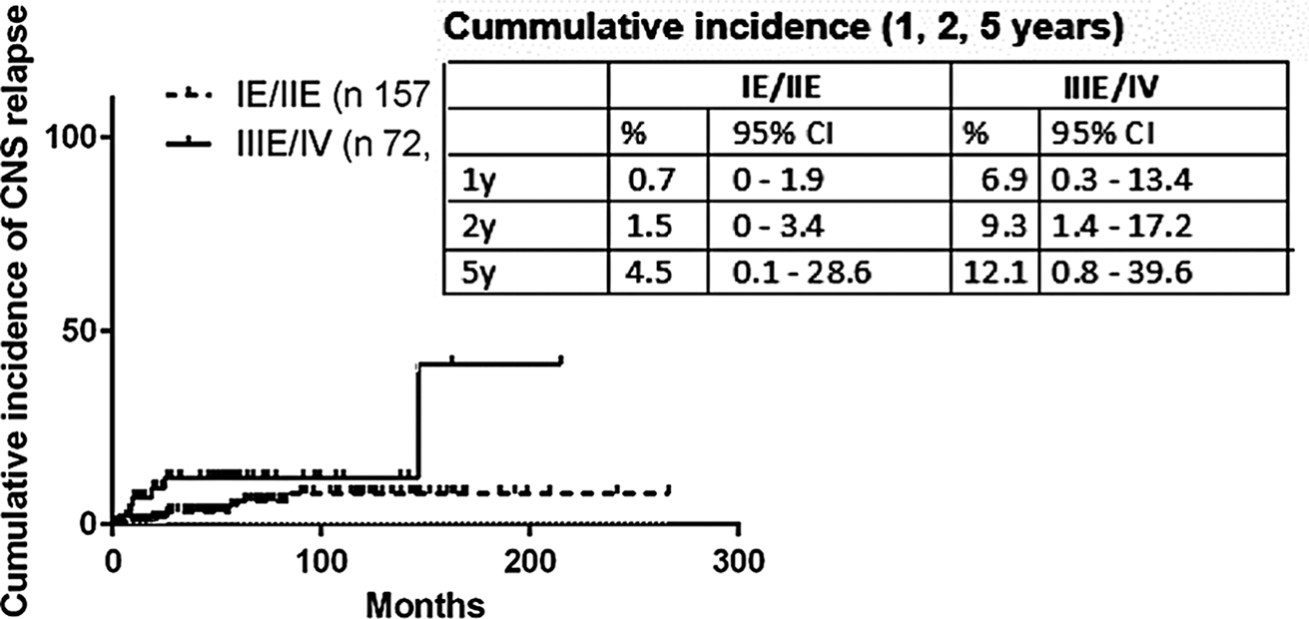
Cumulative incidence of CNS relapse: primary testicular lymphoma (IE/IIE) and advanced T-DLBCL with testicular involvement (IIIE/IV)

Median follow-up of all patients was 51.8 months. Median follow-up of PTL and T-DBCL was similar: 104.3 months (range 0.79–266.8) vs. 72.5 months (range 1.55–215.1), *p* = 0.17. Overall about a third relapsed (*n* = 63; 27.5%; Table 2). Median time to CNS relapse 21.9 months and to systemic relapse 14.7 months (*p* = 0.63). Median time to CNS relapse was longer in PTL (40.5 months) compared to T-DLBCL (14.1 months), yet did not reach significance due to small number of events. The 5-year cumulative incidence of CNS relapse in PTL was lower than in T-DLBCL (4.5 [95% CI, 0.1–28.6%] vs. 12.1% [95% CI, 0.8–39.6%]]; *p* = 0.02; Fig. 1). The rate of systemic relapses did not differ significantly between PTL (*n* = 28) and T-DLBCL (*n* = 21), *p* = 0.7628. The rate of isolated CNS relapses was comparable in both groups (PTL 7 and T-DLBCL 3, *p* = 0.4903). Combined systemic and CNS relapses occurred in four T-DLBCL cases. The distribution of CNS relapse did not differ significantly between PTL and T-DLBCL (*p* = 1.0) with brain parenchyma being the major site of involvement in PTL and T-DLBCL, Table 2. Treatment of CNS +/- systemic relapse was based on high dose MTX 3 g/m^2^ i.v. in 8 patients. R-ICE (rituximab, ifosfamide, carboplatin and etoposide) followed by ASCT (autologous stem cell transplantation) was administered in one patient and another patient received R-ESHAP followed by ASCT. Whole brain radiotherapy without chemotherapy was indicated in one patient. Palliative steroid treatment was administered in one patient and two patients were not treated due to poor performance status.

**Table 2:** Relapses

Relapse	All patients n = 229 (%)	Primary testicular lymphoma n = 157(%)	Advanced disease with testicular involvement n = 72 (%)	P
Lymphoma relapse	63 (27.5)	35 (22.3)	28 (38.9)	
Isolated systemic	49 (77.8)	28 (80.0)	21 (75.0)	0.76
Isolated CNS	10 (15.9)	7 (20.0)	3 (10.7)	0.49
Both	4 (6.3)	0 (0.0)	4 (14.3)	-
Site of CNS relapse				
Parenchymal	11(78.6)	5 (71.4)	6 (85.7)	1.0
Leptomeningeal	2 (14.3)	1(14.3)	1 (14.3)	
Both	1 (7.1)	1(14.3)	0 (0.0)	

**Fig. 2:**
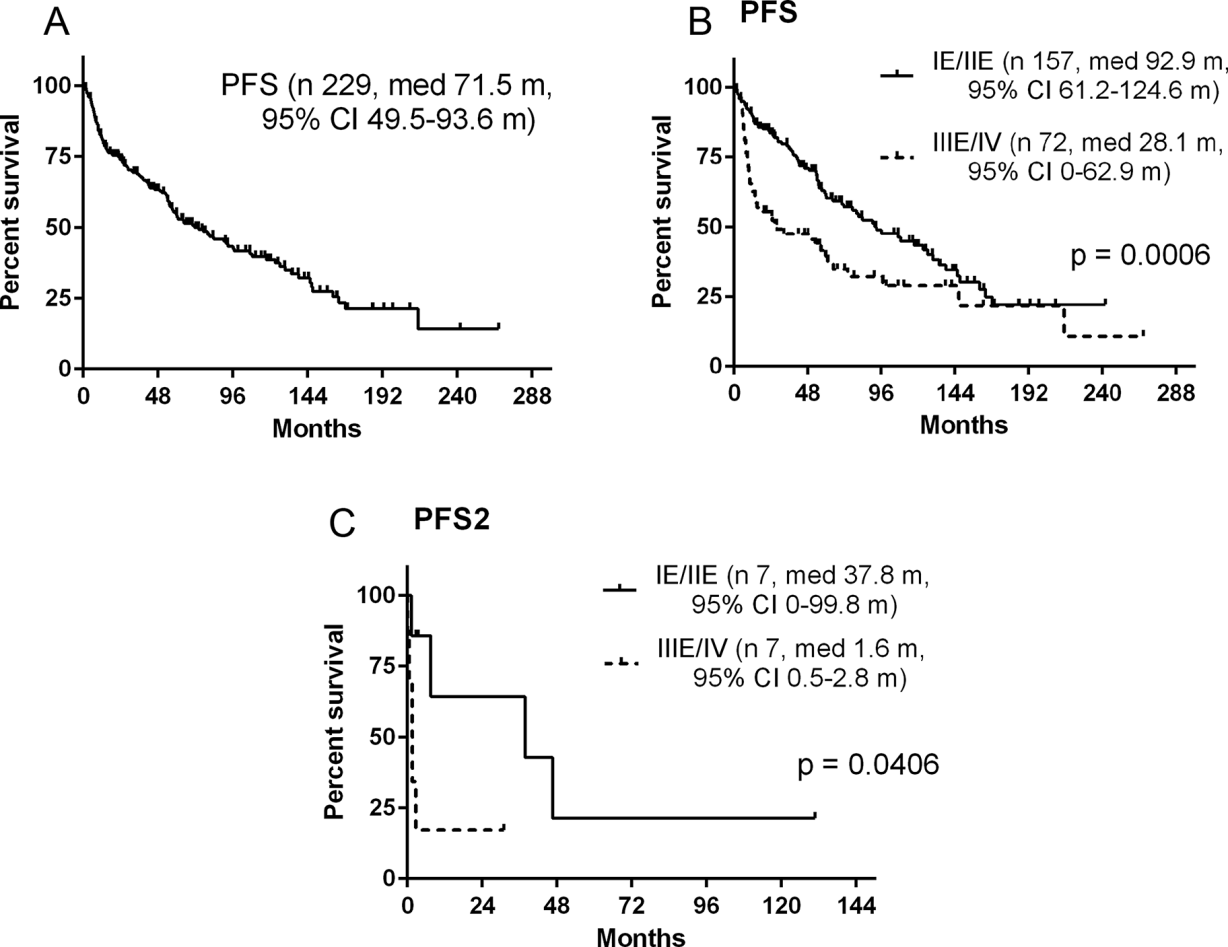
Progression-free survival: all patients (**A**), patients with primary testicular lymphoma and with advanced T-DLBCL (**B**), CNS relapse-patients with primary testicular lymphoma and with advanced T-DLBCL (**C**)

At median PFS of entire cohort 71.5 months (95% CI, 49.5–93.6) the 5-year PFS was 54.8%, Fig. 2A. Median PFS in PTL was significantly longer when compared to T-DLBCL (92.9 months [95% CI, 61.2–124.6] vs. 28.1 months [95% CI, 0–62.9]; *p* = 0.0006; Fig. 2B). Similarly, the 5-year PFS was better in PTL than in T-DLBCL (62.0% vs. 39.5%, *p* = 0.0006). Median follow-up since CNS relapse was 3.3 months (range 0.4-130.8). Median PFS2 from CNS relapse was dismal in T-DLBCL compared to PTL (PFS2 1.6 months [95% CI, 0.5–2.8] vs. 37.8 months [95% CI, 0–99.8]; *p* = 0.0406), Fig. 2C.

In univariate analysis for CNS relapse, we analyzed patients and disease characteristics (presence of B symptoms, age, elevated LDH, ECOG performance status 2–4, International Prognostic Index, IPI of 3–5, kidney and suprarenal involvement, number of extranodal sites ≥ 3) as well as CNS prophylaxis and treatment (orchiectomy, RT, MTX i.t., MTX i.v., both i.t. and i.v. and rituximab based chemotherapy), Supplemental Table_S1. In multivariate analysis, MTX prophylaxis (i.v. and/or i.t.) and testicular RT had no impact on CNS relapse (*p* = 0.49 and *p* = 0.60). Orchiectomy was the single significant factor associated with lower risk of CNS relapse in PTL (HR = 0.11 [95% CI, 0–0.124]; *p* = 0.001). Rituximab based chemotherapy significantly reduced CNS relapse in T-DLBCL (HR = 0.1 [95% CI 0.002–4.70]; *p* = 0.0005) but had no impact in PTL.

Univariate analyses for PFS and OS included: elevated LDH, ECOG performance status 2–4, IPI of 3–5, kidney and suprarenal involvement, number of extranodal sites ≥ 3, age, orchiectomy, rituximab based chemotherapy, MTX prophylaxis i.t., i.v., both i.t. and i.v., RT. Significant risk factors for PFS and OS in univariate analyses were ECOG performance status 2–4, IPI of 3–5 and increasing age in both PTL and T-DLBCL, Supplemental Table_S2. Elevated LDH was associated with inferior PFS in T-DLBCL and with worse OS in PTL and T-DLBCL, Supplemental Table_S2. Involvement of EN sites (≥ 3) were associated with lower PFS in T-DLBCL, Supplemental Table_S2. Rituximab based chemotherapy and RT yield improved PFS and OS in PTL and T-DLBCL, Supplemental Table_S2. Only 5 of 103 patients without RT to contralateral testis relapsed in this site. MTX i.t. prophylaxis was significant for PFS and OS in PTL and combined MTX i.t. and i.v. in advanced disease was significant for PFS and OS, Supplemental Table_S2.

Multivariate analyses for PFS and OS comprised risk factors, prophylaxis and treatment that were significant in univariate analyses: elevated LDH, ECOG performance status 2–4, IPI of 3–5, number of extranodal sites ≥ 3, age, RT, MTX prophylaxis and rituximab based chemotherapy, Supplemental Table_S2. Younger age and rituximab based chemotherapy were significant factors for better PFS and OS in PTL, furthermore RT was associated with better PFS and elevated LDH was unfavorable factor for OS in PTL, Supplemental Table_S2. In T-DBCL, RT was significant for PFS and ECOG performance status 2–4 for OS, Supplemental Table_S2.

**Fig. 3:**
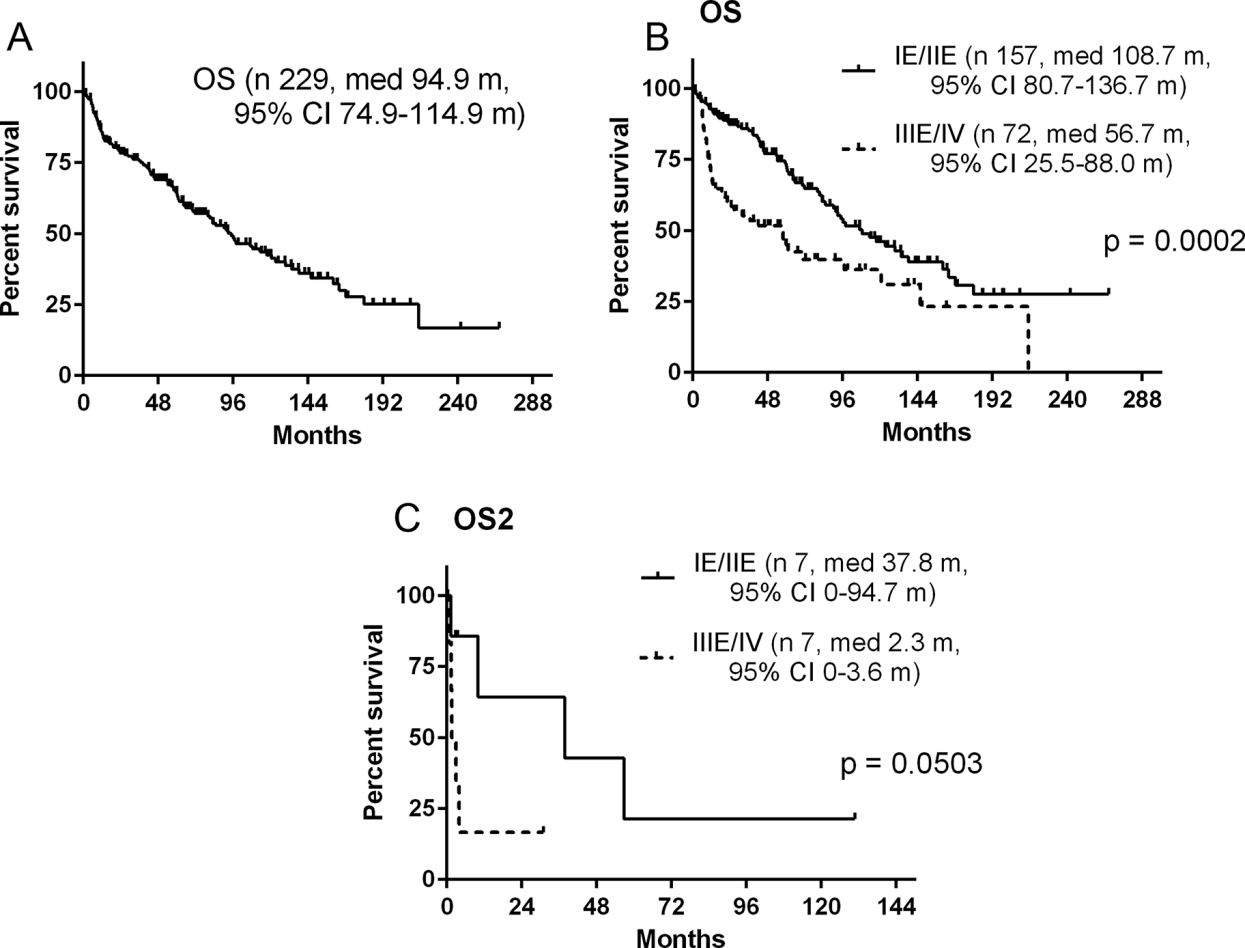
Overall survival: all patients (**A**), patients with primary testicular lymphoma and with advanced T-DLBCL (**B**), CNS relapse-patients with primary testicular lymphoma and with advanced disease (**C**)

Overall 110 (48%) patients died: PTL 68 of 157 (43.3%) and T-DLBCL 42of 72 (58.3%). There was a trend towards a higher death rate in systemic relapses in T-DLBCL vs. PTL, it did not reach a statistical significance (17 [40.5%] vs. 15 [22.0%], *p* = 0.0519). Out of 14 patients with CNS relapse, 8 died due to lymphoma progression in CNS and the difference was not significant between PTL and T-DLBCL (3 vs. 5, *p* = 0.26). Second tumors led to deaths in 7 patients with PTL and in one patient with T-DLBCL (*p* = 0.1514). Lethal treatment toxicities were higher in T-DLBCL (infections 4, CNS hemorrhages 2, renal failure 1, cardiac failure 1) when compared to PTL (infection 1), *p* = 0.0018. Other causes of death not specifically defined were recorded in 12 patients. Cause of death was unknown in 41 patients (34 in PTL, *p* = 0.0005). Median OS of the whole cohort was 94.9 months (95% CI, 74.9–114.9) and the 5-year OS was 64.6%, (Fig. 3A). Median OS in PTL was significantly better when compared to T-DLBCL (108.7 months [95% CI, 80.7–136.7] vs. 56.7 months [95% CI, 25.5–88.0]; *p* = 0.0002), Fig. 3B. The 5-year OS was better in PTL 73.5% than in T-DLBCL 45% (*p* = 0.0002). Median OS2 since CNS relapse was significantly shorter in T-DLBCL compared to PTL (OS2 2.3 months [95% CI, 0–3.6] vs. 37.8 months [95% CI, 0–94.7]; *p* = 0.05), Fig. 3C.

## Discussion

The optimal strategy for CNS prophylaxis in patients with testicular lymphoma is not established and our analysis offers some interesting insights. Orchiectomy represents both diagnostic as well as therapeutic intervention in PTL and bilateral orchiectomy is recommended to prevent lymphoma relapse in contralateral testis. Orchiectomy was used for 94.3% of PTL in our cohort and it was the single significant factor that reduced CNS relapse in these patients, but not in advanced T-DLBCL group. The impact of bilateral orchiectomy on CNS or lymphoma relapse could not be reliably evaluated as it was performed in only a minority of patients with advanced T-DLBCL. Orchiectomy had no impact on PFS and OS in either subgroups.

Contralateral testicular RT did not significantly reduce the CNS relapse. Only 5 of 103 patients without RT of contralateral testis in our cohort relapsed in this location. The IELSG 30 trial presented the efficacy of RT in PTL to prevent the risk of contralateral testicular relapse and suggested to omit RT of retroperitoneal lymph nodes due to the lack of isolated retroperitoneal relapses in patients with stage II [21]. Our data confirmed a positive impact of RT on survival in both subgroups resulting from better control of systemic disease rather than prevention of CNS or contralateral testicular relapse. This observation is in accordance with previously reported data demonstrating a positive impact of RT on survival when combined with rituximab based chemotherapy [4, 16–18].

CNS prophylaxis with MTX produced conflicting results. Despite the fact that 84.3% of all patients received MTX prophylaxis, there was no uniform route of MTX application and finally both groups (PTL and T-DLBCL) were treated with 3 different ways of MTX: intravenous 19.1% vs. 16.6%, intrathecal 40.8% vs. 40.4%, or both 24.2% vs. 27.8%. Moreover, there was a heterogeneity in the doses of MTX i.v. This heterogeneity could cause, that the impact of MTX on CNS relapse failed to prove a statistical significance. Our 5-year cumulative incidence of CNS relapse in PTL (4.5%) is close to the result of Vitolo et al., that used 4 cycles of MTX i.t. and the 5-year cumulative incidence of CNS relapse was 6% in the prospective IELSG10 trial [16]. However, this trial was single arm without a comparative treatment approach. CNS relapses occurred signficantly earlier in T-DLBCL (14.1 months) compared to PTL (40.5 months). In contrast to Kridel et al. [12], where patients with T-DLBCL were more likely to have leptomeningeal recurrences (83%), CNS relapses in our patients occurred predominantly in brain parenchyma in both PTL (71.4%) and T-DLBCL (86%). In the subsequent phase 2 prospective trial IELSG30 trial, Conconi et al. used liposomal cytarabine instead of MTX i.t. and 2 cycles of MTX i.v. and did not report any CNS relapse at a median follow-up of 6 years [21]. As MTX i.v. at a dose of 1.5 g/m^2^ does not achieve sufficient concentrations in the cerebrospinal fluid, i.t. liposomal cytarabine was added to ensure higher and more prolonged cytarabine concentrations in the cerebrospinal fluid. The major drawbacks of both prospective studies in PTL is a small number of enrolled patients and a single-arm design. Moreover, liposomal cytarabine for i.t. application was permanently withdrawn from the market because of manufacturing process issues. Similar to prior reports, our retrospective analyses with a larger cohort failed to confirm a reduced risk of CNS relapse after MTX i.t. and suggests lack of efficacy to prevent brain lymphoma involvement [4, 18]. Orellana et al. did not observe significant difference in CNS relapse rate between i.v. or i.t. routes of MTX administration and testicular involvement was associated with a high risk of CNS relapse (11.3%) [22]. Mannisto et al. on the cohort of PTL and T-DLBCL demonstrated, that i.t. MTX had no impact on CNS relapse and MTX i.v. concomitantly with contralateral irradiation or orchiectomy of the contralateral testis improved prognosis. Survival benefit resulted from better control of systemic disease [13].

Previous data demonstrated that addition of rituximab to chemotherapy in PTL and T-DLBCL improved the control of systemic disease, although, its impact on the risk of CNS relapse was not clearly confirmed [4, 12, 13, 18]. This could be partly explained by the fact, that rituximab based chemotherapy received only 53-64% of patients in these reports [4, 12, 13, 18]. In our cohort, rituximab based chemotherapy was used for 90.3% of T-DLBCL and 80.9% of PTL, as several treating physicians tended to omit rituximab based chemotherapy in localized PTL IE. Addition of rituximab was the only significant factor that reduced the risk of CNS relapse in T-DLBCL but not in PTL group. CNS relapses still occurred in both subgroups, but at a reduced rate. The outcome of CNS relapse is dismal, especially in advanced disease with median survival of 2.3 months. Rituximab based chemotherapy improved the control of systemic disease in PTL and T-DLBCL with a significant impact on PFS and OS in univariate analysis, but in multivariate analysis it was significant for PFS and OS in PTL only. ECOG performance status 2–4 was a significant factor for OS in T-DLBCL but not in PTL in multivariate analysis. Age ≥ 60 years remained a sigificant factor for PFS and OS in multivariate analyses in PTL. RT was significant for PFS and OS in PTL and T-DLBCL. The 5-year PFS (54.8%) and OS (64.6%) in our cohort is better than in other retrospective analyses in PTL and T-DLBCL [4, 12, 13, 18, 19], where the rate of patients on rituximab was lower and the rate of CNS relapses was higher. More favorable outcome of PTL was observed in prospective IELSG10 and IELSG30 trials, where all patients received a uniform CNS prophylaxis and rituximab [16, 21]. Additional point is the retrospective design of our analysis, where various treatment approaches were used. Our analysis has several limitations: the low number of events that reduced the power of our analysis, retrospective design, unknown histological subtype in 44.1% of patients, heterogeneity in treatment doses and frequency, no data on transplantation and pre-CAR-T era. There is an unbalanced distribution among several subgroups of patients, especially with regard to CNS prophylaxis, rituximab use and contralateral testis irradiation.

The CNS International Prognostic Index (CNS-IPI) was developed in the era of rituximab based chemotherapy and it defines a high-risk group with 4–6 risk factors who have a risk of CNS relapse of 10% or higher, although, it does not capture the full spectrum of extranodal sites that are associated with CNS relapse like testis, bone marrow, uterine or breast involvement [23–27]. Additionally, the non-GCB subtype of DLBCL is also associated with a higher risk of CNS relapse [28]. The non-GCB subtype was diagnosed in 34.1% in our cohort, however, the subtype of DLBCL was unknown in 44.1% so the impact of histological subtype on CNS relapse could not be reliably established in our retrospective study, Table 1. According to our univariate analyses, neither factors comprised in CNS-IPI nor three or more extranodal sites or kidney or suprarenal involvement were significantly associated with CNS relapse in T-DLBCL, possibly due to a relatively low number of patients with three or more extranodal sites or kidney or suprarenal involvement, Table 1. The CNS-IPI does not delineate which patients benefit, and which do not, from CNS prophylaxis. Despite CNS prophylaxis and rituximab based treatment in combination with RT, testicular involvement still represents a risk factor for CNS relapse. Identifying CNS lymphoma involvement is an important strategy especially in patients with a high risk of CNS relapse. Recent reports successfully used circulating tumor DNA for detection of CNS lymphoma, risk stratification and treatment guidance [29–32]. Cerebrospinal fluid miRNA can also be used for monitoring of CNS relapse [33].

## Conclusions

This study confirmed a significant favorable impact of rituximab in prevention of CNS relapse in advanced DLBCL with testicular involvement. Methotrexate prophylaxis i.v. or i.t. did not alter the CNS relapse risk. Orchiectomy was the single significant factor associated with lower risk of CNS relapse in localized primary testicular lymphoma. Prognosis of CNS relapse is particularly poor in T-DLBCL. Better systemic control of the disease resulted in improved outcome in patients treated by combination of rituximab based chemotherapy and radiotherapy. Increased age and poor ECOG performance status are risk factors affecting the overall survival.

## Electronic supplementary material

Below is the link to the electronic supplementary material.

Supplementary Material 1

Supplementary Material 2

## Data Availability

Due to the nature of protected health information, data are not available for download.
