# Supplementary material for: Outcome of patients with diffuse large B-cell lymphoma and testicular involvement – real world data
Source: Ann Hematol. 2024 Oct 1;104(1):675–84. doi: 10.1007/s00277-024-06025-y (PMC11868350; doi:10.1007/s00277-024-06025-y)
Supplement: Supplementary file 1 — Supplementary Material 1 [file 277_2024_6025_MOESM1_ESM.docx]

Supplemental Table S1 Univariate and multivariate analysis for OS and PFS

| Univariate analysis | | | | | | |
| --- | --- | --- | --- | --- | --- | --- |
| **Overall survival** |  | | |  | | |
|  | Primary testicular lymphoma  (n=157 patients) | | | Advanced disease  (n=72 patients) | | |
|  | HR | 95% CI | P | HR | 95% CI | P |
| LDH elevated | 2.014 | 1.30-3.85 | 0.0037 | 2.581 | 1.41-4.75 | 0.0026 |
| ECOG 2-4 | 3.263 | 2.76-21.89 | 0.0001 | 2.574 | 1.65-8.03 | 0.0018 |
| IPI 3-5 | 9.678 | 0.87-106.7 | < 0.0001 | 4.41 | 1.48-5.94 | 0.004 |
| Age | 1.054 | 1.028-1.081 | < 0.0001 | 1.064 | 1.024-1.105 | 0.0014 |
| Rituximab+ | 0.3554 | 0.13-0.46 | < 0.0001 | 0.2211 | 0.05-0.99 | < 0.0001 |
| MTX i.t.+ | 0.6605 | 0.34-0.87 | 0.0129 | 1.106 | 0.61-2.05 | 0.7328 |
| MTX i.t.+ i.v.+ | 0.531 | 0.33-1.07 | 0.0835 | 0.3291 | 0.22-0.82 | 0.0127 |
| Radiotherapy+ | 0.5675 | 0.34-0.90 | 0.0177 | 0.3485 | 0.19 – 0.63 | 0.0007 |
| **Progression-free survival** | | | | | | |
|  | HR | 95% CI | P | HR | 95% CI | P |
| LDH elevated | 1.556 | 0.98-2.69 | 0.0595 | 2.314 | 1.33-4.19 | 0.0042 |
| ECOG 2-4 | 3.005 | 2.46-15.38 | 0.0001 | 2.573 | 1.70-7.52 | 0.001 |
| IPI 3-5 | 6.23 | 0.89-43.5 | < 0.0001 | 3.875 | 1.47-5.37 | 0.0031 |
| EN sites ≥ 3 | - | - | - | 1.879 | 1.05-4.39 | 0.0328 |
| Age | 1.044 | 1.021-1068 | 0.00016 | 1.056 | 1.017-1.096 | 0.0047 |
| Rituximab+ | 0.3898 | 0.15-0.51 | < 0.0001 | 0.2344 | 0.05-1.02 | < 0.0001 |
| MTX i.t.+ | 0.5721 | 0.36-0.88 | 0.0132 | 1.268 | 0.73-2.30 | 0.3979 |
| MTX i.t.+ i.v.+ | 0.6038 | 0.37-1.11 | 0.1136 | 0.3231 | 0.22 – 0.77 | 0.0058 |
| Radiotherapy+ | 0.4949 | 0.29-0.75 | 0.0016 | 0.2928 | 0.16 – 0.49 | < 0.0001 |
|  |  |  |  |  |  |  |

| Multivariate analysis | | | | | | |
| --- | --- | --- | --- | --- | --- | --- |
| **Overall survival** | | | | | | |
|  | HR | 95% CI | P | HR | 95% CI | P |
| ECOG 2-4 | 1.8765 | 0.71-4.94 | 0.20267 | 2.5758 | 1.00-6.62 | 0.0494 |
| Age | 1.0441 | 1.02-1.07 | 0.00049 | 1.0395 | 0.99-1.08 | 0.0588 |
| Rituximab+ | 0.4163 | 0.23-0.76 | 0.0046 | 0.4791 | 0.18-1.2 | 0.1381 |
| **Progression-free survival** | | | | | | |
|  | HR | 95% CI | P | HR |  |  |
| Age | 1.0345 | 1.01-1.06 | 0.00298 | 1.0295 | 0.99-1.07 | 0.1268 |
| Radiotherapy + | 0.5843 | 0.37-0.93 | 0.02434 | 0.354 | 0.16-0.77 | 0.00859 |
| Rituximab+ | 0.5071 | 0.29-0.89 | 0.01809 | 0.538 | 0.21-1.40 | 0.20424 |

HR, hazard ratio; CI, confidence interval; ns, not significant; LDH, lactate dehydrogenase; ECOG, Eastern Cooperative Oncology Group; IPI, International Prognostic Index; EN, extranodal;

Not significant for OS on univariate analysis were: kidney/adrenal involvement, number of extranodal sites, orchiectomy and intravenous methotrexate.

Not significant for PFS on univariate analysis were: Kidney/adrenal involvement, orchiectomy and intravenous methotrexate.

Not significant for OS on multivariate analysis were: elevated LDH, number of extranodal sites, testicular radiotherapy, and CNS prophylaxis.

Not significant for PFS on multivariate analysis were: elevated LDH, ECOG, number of extranodal sites, and CNS prophylaxis.
